# Stress, anxiety, emotion regulation and social support in parent‐child dyads prior to and during the onset of the COVID‐19 pandemic

**DOI:** 10.1002/smi.3183

**Published:** 2022-08-05

**Authors:** Audrey‐Ann Journault, Emy Beaumont, Sonia J. Lupien

**Affiliations:** ^1^ Centre for Studies on Human Stress Montreal Québec Canada; ^2^ Research Center Institut universitaire en santé mentale de Montréal Montreal Québec Canada; ^3^ Department of Psychology Université de Montréal Montreal Québec Canada; ^4^ Department of Psychiatry and Addiction Université de Montréal Montreal Québec Canada

**Keywords:** anxiety, COVID‐19, emotion regulation strategies, longitudinal study, mental health, parent‐child dyads, stress

## Abstract

In March 2020, and in order to assess the impact of the COVID‐19 pandemic on stress and mental health in parent‐child dyads using pre‐pandemic measures, we recontacted participants from a 2019 study. A total of 136 dyads of Canadian parents (77% mothers, mean age = 44.48 years/old) and children (63% girls, 77% aged 10–12 years/old and 23% aged 15–17 years/old) completed self‐report measures of perceived stress, anxiety (state/sensitivity) and emotion regulation strategies (cognitive reappraisal/expressive suppression). Children additionally completed measures of co‐rumination and perceived social support from friends, parents, and teachers. Results revealed a significant increase in parents' stress and state anxiety during the pandemic compared to before, but not in their children. Dyads' anxiety sensitivity remained unchanged, as well as parents' use of cognitive reappraisal and expressive suppression. Children showed similar use of cognitive reappraisal, but less expressive suppression and co‐rumination during the pandemic compared to before. Children reported similar perceived social support from all sources over time. Finally, parental and children scores were not significantly correlated at either time. These results suggest that during the onset of the COVID‐19 pandemic, parents and children responded differently in terms of stress, anxiety, and emotion regulation strategies.

## 1 INTRODUCTION

When the COVID‐19 pandemic struck in March 2020, it drastically transformed the daily lives of families across the globe. At the centre of this crisis, the emergency sanitary restrictions and related confinement measures implemented to slow down the spread of the virus generated heightened havoc in the lives of children and parents. In Quebec, Canada, elementary and high schools were closed for 2 months with a variation in the frequency and intensity of non‐mandatory distant learning provided to children. High schools remained closed until the end of the school year in June 2020, and annual government exams at both elementary and high school levels were cancelled. Public venues, including sports facilities and non‐essential services were also closed, leading many parents to lose their jobs or work from home while taking care of their children. In addition, all indoor and outdoor social gatherings were prohibited. These unprecedented disruptions that the pandemic caused to daily life soon became a crucial opportunity for scientists to study the psychological consequences on families (Lupien et al., 2009) as this unique situation could have significant repercussions (Pietrabissa & Simpson, 2020) on the mental health of children and/or their parents.

Over the last 2 years, there has been a rapid growth in the literature concerning the mental health effects of the pandemic in families. Researchers faced many methodological challenges, which led to certain biases in studies (Robinson et al., 2021). Examples of such biases include (1) a lack of pre‐pandemic data among the same participants (in order to adequately attribute the observed individual mental health changes to the pandemic), (2) a lack of self‐reported data in children, and (3) a sole focus on the negative effects of the pandemic.

### 1.1 Longitudinal studies assessing stress and anxiety in children and parents before and during the pandemic

To date, most of the studies that have been published since the World Health Organisation declared COVID‐19 a pandemic on 11 March 2020 used cross‐sectional data, retrospective reports or comparisons to national norms to evaluate how the onset of the pandemic affected the mental health of children and parents. As such, Canadian meta‐analysis found clinically elevated symptoms of anxiety in youth to have doubled compared to *pre‐pandemic estimates in a different group of participants* (Racine, McArthur, et al., 2021), and an American study found the stress of parents (95% mothers) to be greater in May 2020, than before the pandemic, *when assessed retrospectively* (*N* = 433; Adams et al., 2021). However, these methods make it difficult to attribute the mental health changes solely to the pandemic, given that many other factors could modulate mental health across time (Pierce et al., 2020; Van den Bergh & Walentynowicz, 2016). According to Chadi et al. (2022), only a few ongoing longitudinal studies in families that started before the pandemic were able to seize the opportunity to add measures at the onset of the pandemic (the first months following the implementation of government restrictions; e.g.: Achterberg et al., 2021; Barendse et al., 2021; Feinberg et al., 2021; Gadermann et al., 2021; Magson et al., 2021; Racine, Hetherington, et al., 2021; Robinson et al., 2021; Wong et al., 2022). As such, they could adequately infer mental health repercussions of the pandemic because their studies included baseline measures among the same participants. Overall, these studies reported detrimental effects of the pandemic on the mental health of children and parents worldwide. Australian adolescents (*N* = 248, mean age = 14.4 years/old) reported more anxiety (greater effect in girls) during the two first months of the pandemic than 12 months prior to the pandemic (Magson et al., 2021). American children and their parents (*N* = 129 families) also reported a similar increase in anxiety (Feinberg et al., 2021) during the first months of the pandemic compared to before, whereas Chinese mothers reported an increase in stress, as well as an increase in stress and anxiety in 39.1% of their children (*N* = 233, mean age = 12 years/old; Wong et al., 2022). In Canada, a study suggests an increase in anxiety in mothers during the summer of 2020 compared to their clinical levels of anxiety 3, 5 and 8 years prior to the pandemic (*N* = 1301; Racine, Hetherington, et al., 2021). In addition, a recent systematic review and meta‐analysis of 65 studies looking at the change in mental health measures among the same groups before and during the first six months of the pandemic concludes that there exists an overall decrease of mental health in the general population (Robinson et al., 2022). However, this review excluded studies measuring stress and did not evaluate the change in mental health outcomes specific to parents (vs. other adults), while another Canadian study found that parents were more affected during the first months of the pandemic compared to other adults (Gadermann et al., 2021).

Interestingly, findings from other studies demonstrated both detrimental and beneficial effects on children's mental health (Tardif‐Grenier et al., 2021). A study of 385 Canadian teenagers found that 67%–70% experienced a *retrospective* deterioration of mental health in the first two to four months of the pandemic, while 19%–30% of the sample experienced better mental health (Cost et al., 2021). In a similar vein, adverse effects of the pandemic on the mental health of children were found in young Canadian boys, but not in young girls during the two first months of the crisis (*N* = 213, mean age = 5.69 years/old; Browne et al., 2021). A collaborative study from USA, Netherlands and Peru found a decrease in anxiety symptoms of adolescents younger than 13 years/old, but no change in older ones during the first six months of the pandemic, where girls again reported greater symptoms of anxiety than boys (*N* = 1339, 9–18 years/old; Barendse et al., 2021).

### 1.2 The importance of self‐reported data in children

Although results from the abovementioned longitudinal studies are interesting, the majority lack self‐report measures of children's mental health, as most data on children's mental health are obtained by their parents, especially by mothers (Browne et al., 2021; Cost et al., 2021; Feinberg et al., 2021; Gadermann et al., 2021; McArthur et al., 2021). Adult reports of their child's mental health (notably reports from mothers) are known to present discrepancies with reports provided by the child, with over‐or underestimation in adults' reports (Kim et al., 2016; Van Roy et al., 2010). Consequently, longitudinal data including baseline self‐report measures of children's *and* parents' mental health are crucial to accurately documenting any detrimental or beneficial effects of the COVID‐19 pandemic on the mental health of all family members.

### 1.3 How families regulate stress and anxiety also matters

The majority of studies have examined whether the pandemic has negatively affected the mental health of families, but it is also important to consider that negative emotion can be adaptive when facing an acute stressful situation such as the onset of a pandemic (Beesdo et al., 2009; Gross, 2015). Some family members may have adequately coped with these negative emotions and adapted to the crisis. For example, studies have shown that certain families even benefited from this unique opportunity to create stronger family bonds (Achterberg et al., 2021; Evans et al., 2020). The rise in negative emotions such as stress and anxiety during the onset of the pandemic would then be deemed as having less of a “detrimental effect” on the mental health of some families if family members used effective emotion regulation strategies to deal with the stress of the pandemic and gathered social support when needed.

Emotion regulation is defined as “the attempts to influence which emotions one has, when one has them, and how one experiences or expresses these emotions” (Gross, 2015, p. 5). The literature, including meta‐analyses, suggests that the use of proactive emotion regulation strategies (i.e., cognitive reappraisal or gathering for social support) are more efficient in regulating negative emotions and physical responses to emotion‐eliciting stimuli (Aldao et al., 2010), compared to passive, avoidance‐oriented strategies (i.e., expressive suppression or co‐rumination; Diefendorff et al., 2008; Restubog et al., 2020; Schäfer et al., 2017). *Cognitive reappraisal* is the action of ‘changing thoughts and beliefs about the meaning of a stimulus or situation’ (Aldao et al., 2010; Gross & John, 1998; Schäfer et al., 2017, p. 262), whereas *Expressive suppression* is concealing outward displays of emotions (Gross, 2013) or acting for “burying” them inside (Rawana et al., 2014). In terms of repercussions on negative emotions, less frequent use of cognitive reappraisal has been associated with greater depression and anxiety symptoms (Dryman & Heimberg, 2018; Eastabrook et al., 2014; Lanteigne et al., 2014; Nolen‐Hoeksema & Aldao, 2011; Xu et al., 2020), just like more frequent use of expressive suppression has been bidirectionally associated with symptoms of depression and anxiety in adults during the pandemic (Dawel et al., 2021). *Social* *support* is described as the “support accessible to an individual through social ties to other individuals, groups, and the larger community” (Lin et al., 1979, p. 109), whereas *co‐rumination* is the tendency to discuss a problem repeatedly and exhaustively with a relative or friend while focussing on the causes of a problem, its consequences, and the associated negative feelings (Rose, 2002). Perceived social support has protective effects on the mental health of individuals when facing stressful situations (Kawachi, 2001; Khan & Husain, 2010). Despite the restricted social interactions imposed in the first month of the pandemic, the protective effect of social support during the pandemic has been observed across all age groups (Li et al., 2021), and some individuals resorted to online technology as a means of coping through virtual social support (Gabbiadini et al., 2020). On the other hand, instead of regulating negative emotions, co‐rumination can exacerbate them and has been associated with greater anxiety (Carlucci et al., 2018; Spendelow et al., 2017). The use of diverse emotion regulation strategies and their efficacy are closely tied to development and sex. Compared to middle childhood (6–12 years/old), the development of executive functions in adolescence facilitates the use of cognitive adaptative strategies during this period (13–18 years/old; Schäfer et al., 2017), where the efficacy of the strategies used seems to vary across sex and to have various repercussions on mental health outcomes (Ferschmann et al., 2021; Rawana et al., 2014). Furthermore, women tend to co‐ruminate and to rely more on social support than men in adulthood (Sanchis‐Sanchis et al., 2020). In order to gather a deeper insight into how family members dealt with the psychological impacts of the pandemic, it is thus important to assess negative emotions such as stress and anxiety, along with coping strategies such as emotion regulation and social support while considering developmental and sex specificities.

### 1.4 Aims and hypotheses

The present study aimed to better understand the stress and psychological effects of the COVID‐19 pandemic on family members while filling the methodological gaps of the existing literature. We took the opportunity to measure the change in stress and anxiety measures during the onset of the pandemic (March‐April 2020—when emergency measures were high) compared to pre‐pandemic measures (May‐June 2019 and October‐November 2019) in a cohort of children and their parents (mothers *or* fathers) enroled in an ongoing study. A second objective was to examine the changes in emotion regulation strategies and perceived social support of family members at both periods. When the study was launched in March 2020, no precise pre‐determined hypothesis could be gathered from the literature as this pandemic was the first of the 21^st^ century. Therefore, the present study was descriptive and exploratory (Gaus, 2015), aiming to generate new hypotheses on how the COVID‐19 pandemic affected family members' stress and mental health. However, between the launch of the present study and the publication of this paper, the literature has allowed us to hypothesise that exposure to the pandemic will negatively affect mental health measures, especially in parents and in females.

## 2 METHODS

Data from this project is embedded in the primary research project entitled: *My Anxiety or Your Anxiety? Associations between psychological and biological markers of stress and anxiety in children, their parents and teachers* (MATA) approved by the Research Ethics Board of the Centre intégré universitaire de santé et de services sociaux de l’Est‐de‐l’Île de Montréal, Québec, Canada in April 2019. The primary project aimed at understanding the nature and strongest predictors of anxiety in children and adolescents. The study ran in 2019 and received a second ethics approval in March 2020 so that the same measures were recollected during the onset of the pandemic. Anonymised data, preregistrations of primary analyses before any analysis was conducted (DOI: 10.17605/OSF.IO/8MRVX), SPSS syntax, a complete list of the measures taken in the primary MATA project, figures and supplemental materials are available on the Open Science Framework (https://osf.io/4jdvw/ data).

### 2.1 Participants

A total of 272 French‐speaking children and parents (136 dyads) from private (*n* = 6) and public schools (*n* = 6) from a northern region of Montreal, Québec, Canada, participated in this study. Dyads comprised of 50 boys and 86 girls (77% aged 10–12 years/old at T1 [mean age = 11.25, SD = 0.53] and 23% aged 15–17 years/old at T1 [mean age = 16.24, SD = 0.48]), and 31 fathers and 105 mothers (mean age at T1 = 44.48 years/old, SD = 5.99). Regarding sample demographic characteristics, 54% of children attended a public school and 10% reported to have received a medical diagnosis for mental health disorders (all diagnoses combined) in pre‐pandemic measures. Children were involved in extracurricular pre‐pandemic activities (*M* = 1.43 per week; SD = 1.44), and 42% of children had discussions about the pandemic (2–5 times/day) during the onset of the pandemic. At the time, when children were asked how worried they were about the following on a scale from 1 (not worried) to 7 (excessively worried; see Supplemental material), children were more worried about the health of their parents (*M* = 4.05, SD = 1.81) or a close one (*M* = 4.64, SD = 1.63). They also had a greater concern about the end of their school year that had been put on hold (*M* = 4.18, SD = 2.09) than about their health (*M* = 2.76, SD = 1.54), the lack of essential supplies (*M* = 2.33, SD = 1.52) or the possibility that their parents may lose their job (*M* = 3.09, SD = 1.78). When children were asked “To what extent has your stress been caused by the pandemic on a scale from 1 (not at all) to 10 (a lot)?”, the mean score was 3.69 (SD = 1.98). In parents, 81% identified as White, 61% reported a pre‐pandemic yearly family income above 100K CAD$, 55% had completed at least a bachelor's degree, 80% were married or common‐law partners and 15% reported having a medical diagnosis of anxiety disorder. During the onset of the pandemic, 47% of parents were working with the public (health system or essentials workers) and 10% had a medical condition putting them at high risk to contract the severe form of the COVID‐19 disease.

### 2.2 Procedure

**FIGURE 1:**
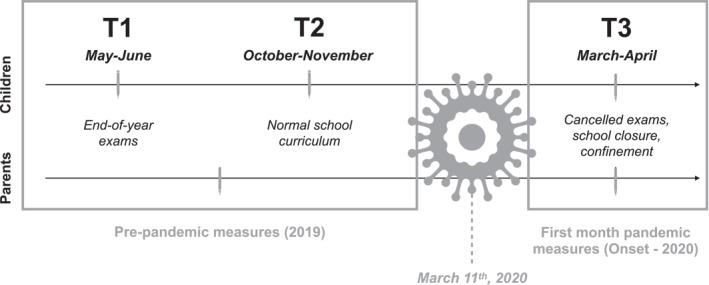
Representation of the procedure and the measures collected as a function of time

**FIGURE 2:**
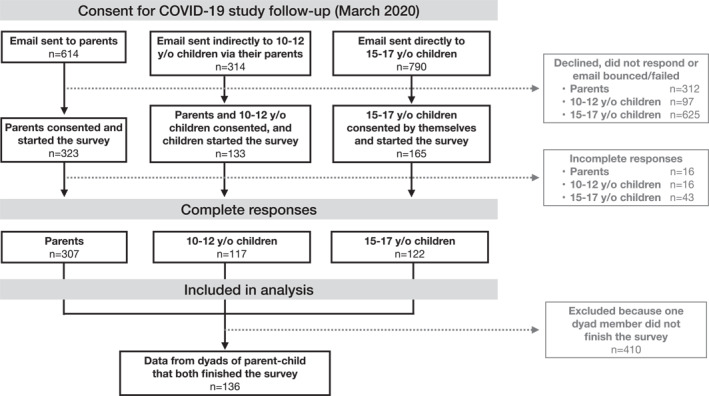
Flow of participants leading to the final sample

Participants were first recruited through schools in 2019, and were recontacted by email during the onset of the pandemic in Canada (Figure 1). All dyads who agreed to participate provided informed consent. Parental consent and children's assent were obtained for children younger than 14 years of age. Data was collected twice in children and parents before the pandemic (using paper/pencil or online questionnaires) and once during the first month following the implementation of government restrictions to slow down the spread of the virus in Quebec (using Qualtrics online versions of the same questionnaires). A flow diagram of participation is presented in Figure 2. In brief, 53% of the parents, 42% of 10–12 years/old children and 21% of 15–17 years/old children who participated in the primary research project continued to take part in the pandemic data collection. Chi‐squared and *T*‐tests performed on the demographic and all outcome variables at baseline revealed no major significant differences for dyads who opted out of the study, except that these parents had a slightly lower level of education (*p* = 0.01) while the children were more likely to be male (*p* = 0.009). Furthermore, these children reported more test anxiety prior to the pandemic (*p* < 0.001).

### 2.3 Measures

**Demographics.** Demographic data (sex, socioeconomical status, ethnicity, etc.) were gathered in the first pre‐pandemic measure in both children and parents. Factual data on participants' adjustment to the pandemic (worries, frequency of discussion on the matter, etc.) were gathered using child and parent versions of an in‐house COVID‐19 questionnaire (see supplemental material to view demographic and COVID‐19 items).

For the current study, only the following pre‐pandemic measures from the primary project were reconducted during the onset of the pandemic (Table 1).

**TABLE 1:** List of all the outcomes measured in each dyad member and at each time point

	Children			Parents	
Measured outcomes	*T1*	*T2*	*T3*	*T1* and/or *T2*	*T3*
Perceived stress	X	X	X	X	X
State anxiety	X	X	X	X	X
Anxiety sensitivity	X	X	X	X	X
Cognitive reappraisal	X	X	X	X	X
Expressive suppression	X	X	X	X	X
Co‐rumination		X	X		
Social support—friends	X	X	X		
Social support—parents	X	X	X		
Social support—teachers	X	X	X		

**Stress.** The French version of the *Perceived Stress Scale* (PSS; Cohen et al., 1983; Lesage et al., 2012) contains 14 items asking responders to estimate how frequently they had stress‐related feelings or thoughts in the past month on a scale from 0 to 4 (0 = “never”, 4 = “very often”). The scores vary from 0 to 56, with higher scores indicating greater perceived stress in the past month. The reliability coefficient of the questionnaire is 0.85 (Cohen et al., 1983) and of *ω* = 0.89 in our sample. The *Perceived Stress Scale for Children* (White, 2014), an adapted version for children aged 5–18 years, contains 13 items on stress‐related feelings and thoughts of the last week that are rated on a scale of 0–3 (0 = “never”, 3 = “a lot”). We translated the questionnaire from English to French using a double‐blind translation technique and found a reliability coefficient of *ω* = 0.75 in our sample.

**Anxiety.** The present study used two different scales from the primary research project to measure anxiety. First, to measure state anxiety, we used a subscale of the *State‐Trait Anxiety Inventory‐Revised for Adults* (*STAI‐Y*; Spielberger et al., 1983) that contains 20 items assessing momentary anxious states in participants. The items ask how respondents feel “*right now, at this moment*” on a 4‐point Likert scale. The scores vary from 20 to 80, where a higher score represents a greater level of state anxiety. The French version of this inventory has been validated and revealed a reliability coefficient of 0.94 and 0.86 for state anxiety in women and men, respectively (Gauthier & Bouchard, 1993). In children, state anxiety was measured using the French adapted version for children aged 9–12 years: the *State‐Trait Anxiety Inventory for Children* (STAI‐C; Spielberger et al., 1983). Items are rated on a 3‐point Likert scale and thus, the scores vary from 20 to 60, where a higher score indicates greater level of state anxiety. The French version of this inventory has been validated (Turgeon & Chartrand, 2003) and we found a reliability coefficient of *ω* = 0.88 for state anxiety in our sample.

Second, *Anxiety Sensitivity Index* (*ASI*; Reiss et al., 1986) contains 16 items with a 5‐point Likert scale (0 = “very little”, 1 = “a little”, 2 = “some”, 3 = “much”, and 4 = “very much”) to assess anxiety sensitivity in adults. Anxiety sensitivity is the fear of anxiety‐related bodily sensations due to beliefs that these sensations will lead to harmful outcomes such as physical illness, social embarrassment, loss of control and mental incapacitation (Reiss & McNally, 1985). The scores vary from 0 to 64, where higher scores represent a greater level of anxiety sensitivity. We found a reliability coefficient of *ω* = 0.91 in our sample. The adapted version for children ages 9–13 years old, the *Childhood Anxiety Sensitivity Index* (CASI; Stassart & Etienne, 2014), contains 18 items with a 3‐point Likert scale (1 = “not at all”, 2 = “a little”, and 3 = “a lot”). The scores vary between 18 and 54, where a higher score reflects greater anxiety sensitivity. The reliability coefficient found in our sample was *ω* = 0.89.

**Emotion regulation strategies**. *Emotion Regulation Questionnaire* (Christophe et al., 2009) contains 10 items that evaluate the use of two emotion regulation strategies in responders: cognitive reappraisal and expressive suppression. Cognitive reappraisal refers to the cognitive process by which an emotion‐eliciting situation is reinterpreted to mitigate or enhance its emotional impact (Gross & John, 2003; Lazarus & Alfert, 1964). On the other hand, expressive suppression involves an attempt to inhibit or hide the expression of an ongoing emotion (Gross & John, 1998). Responders rate each item on a scale from 1 to 7 (1 = “not at all”, 7 = “absolutely”). Scores vary from 6 to 42 for cognitive reappraisal and from 4 to 28 for expressive suppression, where higher scores represent greater use of each emotion regulation strategy, respectively. The reliability coefficient of the French version of the questionnaire is 0.76 for cognitive reappraisal and 0.72 for expressive suppression (Christophe et al., 2009). We found respective reliability coefficients of *ω* = 0.90 and *ω* = 0.80 in our sample. The *Emotion Regulation Questionnaire for Children and Adolescents* (ERQ‐CA; Gosling et al., 2018), is an adapted version for children aged 8–16 years, also contains 10 items that are rated on a 5‐point Likert scale (1 = “strongly disagree”, 2 = “disagree”, 3 = “half and half, 4 = “agree” and 5 = “strongly agree”). Scores vary from 6 to 30 for cognitive reappraisal and from 4 to 20 for expressive suppression. The reliability coefficient of the French version of the questionnaire for our sample were *ω* = 0.80 and *ω* = 0.76. *Co‐rumination questionnaire* (CRQ; adapted 9‐item version (Arroyo, 2013) of the original 27‐item questionnaire (Rose, 2002) measures the tendency of the responder to discuss a problem repeatedly and exhaustively with a friend while focussing on the causes of a problem, its consequences, and the associated negative feelings (Rose, 2002). Each item in the questionnaire was rated on a scale ranging from 1 to 5 (1 = “not at all true”, 5 = “really true”). Participants' total scores varied from 9 to 45, where a higher score indicated greater co‐rumination. Compared to the reliability of the original 27‐item version (*α* = 0.96; Rose, 2002), the short 9‐item version had excellent reliability (*α* = 0.91; Arroyo, 2013). Our research team used a double‐blind translation technique to translate the questionnaire from English to French, and we found a reliability coefficient of *ω* = 0.88 in our sample. Co‐rumination data were available only in children, but not parents of the current study and exclusively at T2 (the second pre‐pandemic measure) and T3 (onset of the pandemic).

**Social** **support**. *The Child and Adolescent Social* *Support* *Scale* (Malecki & Demary, 2002) contain four subscales of 12 items each to measure perceived social support from different sources: parents, teachers, friends, and classmates. In this study, only the first three subscales were used. Responders were asked to rate the frequency of each item using a 5‐point Likert scale from 1 to 5 (1 = “never”, 5 = “always”). Scores of perceived social support for each subscale range from 12 to 60, where a higher score represents more frequent perceived social support. Reliability coefficients of the different subscales ranged from *ω* = 0.87 to *ω* = 0.92 in our sample. Social support data were available only in children of the current study.

### 2.4 Data analysis

Analyses were performed with SPSS 26.0. As all children provided data at T1 and T2 in the pre‐pandemic measures, their data at T3 (onset of the pandemic measure) was compared with the two pre‐pandemic time points. On the other hand, as parents had the choice to provide data at T1 and T2 or only at T1 or T2 in the pre‐pandemic measures, we computed a pre‐pandemic baseline measure for parents using only the first parental score provided at either time (T1 or T2). Data of parents at T3 was thus compared to this one pre‐pandemic baseline score. This was done to maximise the sample size and statistical power. Skewness and kurtosis were below 1 for most variables and below 2 and 7 (Curran et al., 1996) for state anxiety (children) at T1 and T3, state anxiety (parents) at baseline and T3, social support of friends (children) at T1 and T3, and anxiety sensitivity (parents) at baseline and T3.

Repeated measures ANOVAs were conducted to examine differences between time points for each mental health measure while examining the effect of sex. In children, we conducted mixed ANCOVAs to simultaneously examine differences between time points for each mental health measure and the effect of sex while adjusting for school level (elementary vs. high school). The analysis included the school level variable as a covariate because data from the primary research project included two youth age groups: middle childhood children (10–12 years/old) and adolescents (15–16 years/old). We are aware that these two groups differ in stress, anxiety and emotion regulation strategies because of their developmental stage, as discussed in the introduction. However, the focus of the current study was not on developmental differences and the sample size would not permit to examine this concept. We still adjusted for school level (age) in the analysis for two reasons: (1) to examine the effects of the pandemic on mental health measures in our youth sample beyond developmental effects and (2) to control for the great disparity found in the sample distribution between elementary and high school children. Removing high school children from the sample led to the same results. An alpha level of α = 0.01 was used to prevent type I error inflation. Finally, simple Pearson correlations were conducted between the mental health scores of parents and their children. This was done at baseline and again during the onset of the pandemic.

## 3 RESULTS

Statistical indices resulting from the repeated measures ANOVA and mixed ANCOVAs (including *p* values and η^2^_p_) are presented in Table 2 for children and in Table 3 for parents. Estimated marginal means by sex and indices for correlations are detailed in the supplemental material. Main effects of time and sex are presented in the following sections as no TIME × SEX interaction effects were found for none of the variables.

**TABLE 2:** Mixed ANCOVA analyses for differences in children's outcomes across time and by sex, when adjusting for school level

	ANCOVA—time			ANCOVA—sex		
Measure	F	*p*	η^2^_p_	F	*p*	η^2^_p_
Perceived stress (PSS‐C)	3.29	0.042^†^	0.028	10.15	**0.002**	0.080
State anxiety (STAI‐C1)	2.14	0.122	0.018	16.07	**<0.001**	0.122
Anxiety sensitivity (CASI)	0.93	0.388	0.008	12.77	**0.001**	0.100
Cognitive reappraisal (ERQ‐CA)	1.23	0.295	0.011	0.19	0.661	0.002
Expressive suppression (ERQ‐CA)	5.93	**0.004**	0.050	0.48	0.490	0.004
Co‐rumination (CRQ)	21.37	**<0.001**	0.158	1.90	0.171	0.016
Social support—friends (CASSS‐fr)	1.23	0.293	0.011	4.92	0.029	0.041
Social support—parents (CASSS‐pr)	2.01	0.139	0.017	0.10	0.753	0.001
Social support—teachers (CASSS‐te)	1.17	0.312	0.010	0.04	0.840	0.000

*Note*: *p*‐values in bold indicate statistically significant mixed ANCOVAs at the 0.01 level. ^†^ indicates a significant effect of the covariate at the 0.01 level. Degrees of freedom were of (1,114) for Co‐rumination and varied from (2,218) to (2,232) for all other outcomes. The F values reported are Greenhouse‐Geisser.

**TABLE 3:** Repeated measures ANOVA analyses for differences in parents' outcomes across time and by sex

	ANOVA—time			ANOVA—sex		
Measure	F	*p*	η^2^_p_	F	*p*	η^2^_p_
Perceived stress (PSS)	46.97	**<0.001**	0.273	11.99	**0.001**	0.088
State anxiety (STAI‐Y1)	25.56	**<0.001**	0.171	5.86	0.017	0.045
Anxiety sensitivity (ASI)	4.05	0.046	0.032	4.19	0.043	0.033
Cognitive reappraisal (ERQ)	1.57	0.213	0.012	8.90	**0.003**	0.067
Expressive suppression (ERQ)	0.25	0.618	0.002	1.03	0.312	0.008

*Note*: *p*‐values in bold indicate statistically significant repeated measures ANOVAs at the 0.01 level. Degrees of freedom varied from (1,124) to (1, 125) for all outcomes. The F values reported are Greenhouse‐Geisser.

### 3.1 Stress and anxiety

**Children.** Perceived stress, state anxiety and anxiety sensitivity did not differ significantly across the three time points in children when adjusting for school level. Moreover, perceived stress, state anxiety and anxiety sensitivity differed across sex, where girls reported higher scores on each construct compared to boys.

**FIGURE 3:**
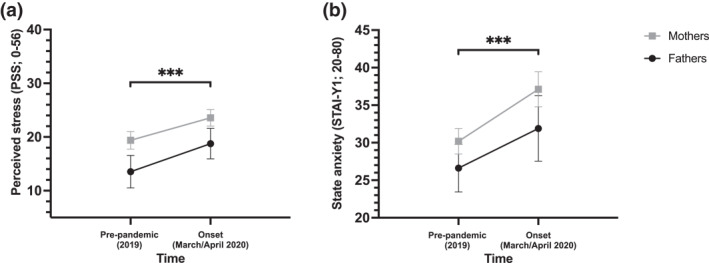
Significative changes in time and by sex for parents' outcomes. 95% confidence intervals of each time point's estimated marginal means by sex for (a) perceived stress in the past month in parents (b) state anxiety in parents. **p < 0.01, ***p < 0.001

**Parents.** Perceived stress and state anxiety differed significantly across the two time points in parents (Figure 3). Pairwise comparisons indicated that parents perceived significantly more stress and experienced greater states of anxiety during the onset of the pandemic than at baseline. Moreover, perceived stress differed across sex, where mothers perceived significantly more stress than fathers. State anxiety did not differ across sex. Anxiety sensitivity did not differ significantly across time, nor sex.

### 3.2 Emotion regulation

**FIGURE 4:**
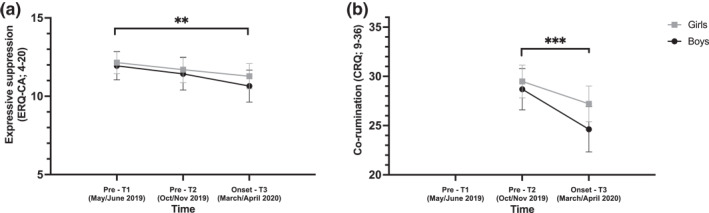
Significative changes in time and by sex for children's outcomes. 95% confidence intervals of each time point's estimated marginal means by sex for (a) expressive suppression in children and (b) co‐rumination in children when adjusting for school level. ** = *p* < 0.01, *** = *p* < 0.001

**Children.** The frequency of cognitive reappraisal did not differ across the three time points nor sex when adjusting for school level. The frequency of expressive suppression differed significantly across time, but not sex when adjusting for school level. Pairwise comparisons indicated that expressive suppression was less frequent during the onset of the pandemic (T3) than at the first baseline measure (T1; Figure 4). No significant difference was found between T1 and T2 nor T2 and T3. Co‐rumination differed significantly across time when adjusting for school level. Pairwise comparisons indicated that children co‐ruminated significantly less during the onset of the pandemic (T3) than at T2 (Figure 4). Co‐rumination did not vary across sexes.

**Parents.** The frequency of cognitive reappraisal and expressive suppression did not differ across the two time points. Cognitive reappraisal was most frequently used by mothers than fathers, while expressive suppression did not differ across sex.

### 3.3 Social support

**Children.** Perceived social support from parents, teachers and close friends did not differ across the three time points nor sex when adjusting for school level.

### 3.4 Correlations between parents' and children's scores

No significant correlation was found between the mental health scores of children and parents prior to nor during the onset of the pandemic.

### 3.5 Exploratory analysis

**FIGURE 5:**
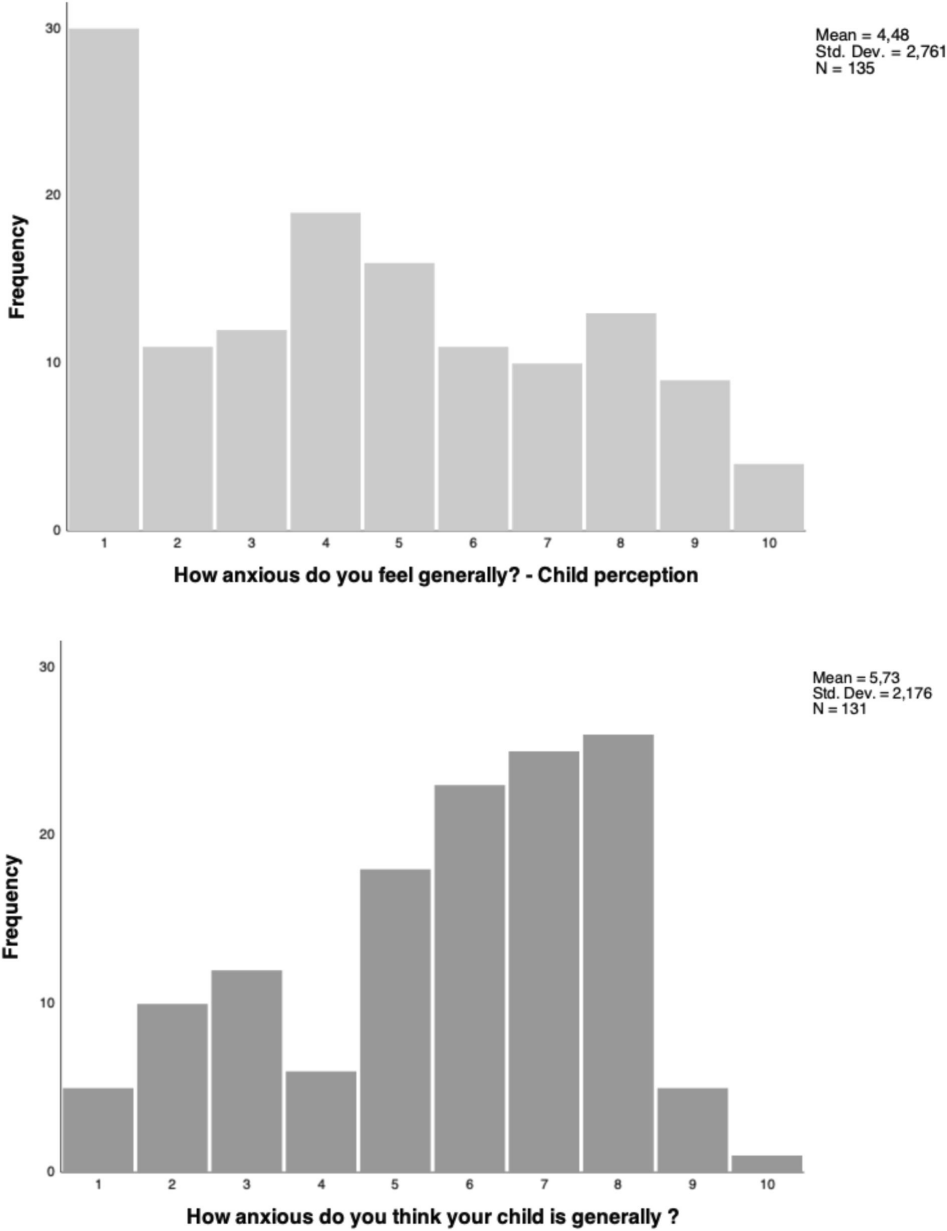
Comparison of the distribution of child's and parent's perception of the child's tendency towards anxiety on a scale from 1 (very little) to 10 (extremely)

To better explain the absence of a relationship between children and parents' reports, we compared the parent's and child's perceptions of the child's general tendency to be anxious. This perception was assessed with a single item from the demographic questionnaire administered prior to the pandemic. Parents were asked “How anxious do you think your child is generally on a scale of 1–10 (1 = ‘very little’, 10 = ‘extremely’)?” and their children were asked “How anxious are you generally (1 = ‘very little’, 10 = ‘extremely’)?”. The correlation between parents’ and children's scores was *r* = 0.44, *p* < 0.01. This shows that children's and parents' perceptions are positively and moderately associated, so that when the parent perceives their child to be anxious, the child also has the perception to be anxious himself. However, the distribution of scores (Figure 5) suggests that parents perceived their children to be more anxious than the children themselves.

## 4 DISCUSSION

This study aimed to add to the existing literature on the effect of the onset of the COVID‐19 pandemic on the stress and mental health of children and parents. This was done by examining the changes in stress, anxiety, emotion regulation and social support in parent‐child dyads using longitudinal self‐report data obtained before the COVID‐19 pandemic and during the first month of the pandemic.

Regarding the change in stress and anxiety levels of family members at the onset of the pandemic, our results are only partially consistent with our hypothesis and previous findings reporting detrimental effects of the pandemic on the mental health of children and parents. Here, we found that the first month of the pandemic did not affect children's levels of perceived stress and state anxiety but was associated with increased perceived stress and state anxiety in parents. This time effect found in parents explained a significant part of the variance (27% and 22% respectively). In addition, we found no effect of the pandemic on measures of anxiety sensitivity in children or parents, and parents' and children's stress and anxiety scores were not associated with each other before or during the first months of the pandemic.

The absence of a time effect on perceived stress and state anxiety in children is in contrast with studies performed in various countries that found a deterioration in children's mental health during the onset of the pandemic (Browne et al., 2021; Cost et al., 2021; Feinberg et al., 2021; Magson et al., 2021; Newlove‐Delgado et al., 2021). Different factors could account for this discrepancy, one being the respondent assessing the child's mental health. Previous studies used parental reports of the child's mental health, while in our study, we directly asked children about their mental health. Studies show that parents tend to overestimate their child's psychological distress, due to their own distress at the time of assessment (Abate et al., 2018; Kassam‐adams et al., 2006; Kelley et al., 2017). The comparison between the child's and parent's perception of the child's general tendency to be anxious supports the hypothesis of an overestimation bias of children's stress and anxiety when using parental reports. This could explain why the current study found no change on these constructs contrary to previous findings. Moreover, this bias would also explain why associations between children's and parents' mental health were found in studies using parental reports of children's mental health (Fitzpatrick et al., 2021; Whittle et al., 2020; Yeasmin et al., 2020), but not in the current study, despite assessing mental states of children using the adapted versions of the same questionnaires. This finding underlines the importance of using children's self‐report data in longitudinal studies, rather than just parental reports to understand the changes in mental health states of children over time.

On the other hand, the significant increase in perceived stress and state anxiety in parents during the first month of the COVID‐19 pandemic is in line with our hypothesis and is consistent with previous findings. National surveys reported that the pandemic's emergency restrictions increased parents' caregiving responsibilities towards their children, “along with concerns about balancing child care, homeschooling and work” (Statistics Canada & Leclerc, 2020, p. 3). In this context, a certain amount of stress and anxious responses from parents could have been adaptative to prepare their bodies to overcome these new challenges (Lupien et al., 2007; Selye, 1998). However, the current study cannot speak of the gravity of the stress and anxiety experienced by family members due to the pandemic as we used validated scales that do not allow to identify if the intensity reaches the clinical cut‐offs of mental health disorders. Qualitative data and assessment of the emergence of clinical manifestations of mental health disorders would be helpful to gain a deeper understanding of how this increase of stress and anxiety experienced by parents during the onset of the pandemic had repercussions on their daily lives.

Regarding the changes in emotion regulation strategies over time, we found that children reported using less expressive suppression and co‐rumination behaviours during the onset of the pandemic. Here, the time effect explained 5% and 17% of the variance in expressive suppression and co‐rumination, respectively. Cognitive reappraisal and social support in children did not change over time. In contrast, parents used similar emotion regulation strategies before and during the pandemic, despite an increase of their stress and anxiety. Again, the emotion regulation scores of children and parents were not associated, either before the pandemic or during the first months of the crisis.

The diminution of expressive suppression and co‐rumination behaviours in children, both inefficient strategies to regulate emotions, could be explained by the confinement measures, which led to school closures. Considering that our sample was composed mainly of families that are financially comfortable and not particularly at‐risk, and that children had spent significantly more time at home during the first month of the worldwide crisis, this experience may have allowed parents and children of our sample to increase the quality of their relationship (Achterberg et al., 2021; Wong et al., 2022). In turn, this could have led parents to pay more attention to their children's emotions and to invite them to talk about their feelings. This factor could explain why children suppressed less emotions than before the pandemic. To test this hypothesis, future studies could compare whether children express their emotions differently across varying contexts (i.e., home vs. school). In their paper, Greenaway et al. (2018) underline the importance of considering contexts when studying emotions and provide practical recommendations to do so.

In a similar vein, it is possible that children had less of a tendency to co‐ruminate because they were at home and not physically at school with their friends. Although children have maintained social relationships with friends while engaged in distant learning, a study has shown that amongst various co‐rumination modalities (in‐person, by text, by social media), most co‐rumination occur in‐person (Battaglini et al., 2021). Finally, given that school is a major source of stress for children (Högberg et al., 2020), they might have had fewer tendency to co‐ruminate about during the onset of the pandemic since they were no longer facing school requirements and challenges. Findings from Li et al. (2022) also support this by suggesting that during the pandemic, 66.7% of Chinese youth experienced partial to overall strong positive changes in many life domains, which could also lead to fewer common pre‐pandemic tendency to co‐ruminate about.

Altogether, the onset of the pandemic appears to have decreased the use of unhealthy emotion regulation strategies in children, without having affected the perceived social support that they received. This might seem contradictory to previous findings (Chadi et al., 2022), but it can be explained by the age of the children participating in the present study. Our sample consisted of mostly middle‐childhood children (10–12 years/old) and only a minority of adolescents (15–17 years/old). Although the analyses were adjusted for this age difference, a systematic review found younger youth to be less impacted by the mandatory decrease of social interactions during the onset of the pandemic compared to adolescents (Panchal et al., 2021). In addition, the burden of this global crisis might have been lighter for children at the onset of the pandemic, compared to their parents since children were deemed to be at low risk to develop the severe form of the COVID‐19 illness during the first months of the pandemic.

While parents' stress and anxiety increased during the first month of the pandemic, they used similar emotion regulation strategies compared to before the pandemic, possibly because the use of emotion regulation strategies is mostly stable through adulthood (Allen & Windsor, 2019). Alternatively, even though the load or intensity of stressors in the parents' environment might have increased (Adams et al., 2021), the nature of the “shift” in this environment might have been less drastic than for children. Compared to children who's school was on break, most parents continued to work and interact with colleagues (virtually or in‐person), which still implies external pressure to regulate emotions, similar to before the pandemic. Future experimental studies should examine if the manipulating social contexts can influence the choice of emotion regulation strategies.

Our study adds to the emerging literature on the psychological effects of the pandemic on families' mental health. It benefits from the joint assessment of negative emotions and emotion regulation strategies in dyads of children and parents, and the fact that it is reliant on baseline measures among the same participants rather than on retrospective recall of pre‐pandemic experiences. The use of data from mothers as well as fathers with a reasonable proportion of boys in the sample group of children is another strength of this study.

Some limitations need to be recognized when interpreting the findings, namely the underrepresentation of families from lower socioeconomic status and various ethnicities, the low response rate between measurement time (although we documented and verified the minor impact of such attrition on the results), the potential bias in study participants due to the volunteering sampling method and the fact that the sample is relatively small. Future studies should pay particular attention to the repercussions of the pandemic on the mental health of at‐risk communities. The burden of the pandemic may have been greater for those individuals who were already vulnerable before the onset of the COVID‐19 pandemic. Another limitation is the lack of assistance from research assistants for children 10–12 years of age when completing the online questionnaire from home during the pandemic. This was unavoidable given the sanitary measures, but some items might have been misunderstood, even though all the scales used in this study have been validated in children of this age group. Finally, while we were able to study the effects of the onset of a health crisis on the stress, anxiety, emotion regulation and social support of parent‐child dyads, this study cannot report on the long‐term repercussions of the pandemic on the mental health of family members. This study found no change in children's mental health measures and previous studies have found little changes. However, it is possible that the disruptions that children faced in their lives could have more salient mental health effects in the long‐term (Fegert et al., 2020). Findings from the McArthur study (2021) suggest that the strongest predictors of adolescents' mental health during the onset of the pandemic when adjusting for pre‐pandemic mental health states were more proximal (e.g., screen time, sleep time, connectedness to parents) than distal (e.g., socioeconomic status, parents' mental states, relation with peers, etc.). However, in the long run and with the maintenance of emergency confinement measures implemented during the pandemic, anxiety and stress experienced by parents were likely to have increased and eventually spilled over to their children (Almeida et al., 1999; Lupien et al., 2000; Wethington, 2000). This is shown in a recent study (Robertson et al., 2021), and given the well‐known detrimental effects of cumulative exposure to stress hormones on cognitive and emotional processes (Lupien & McEwen, 1997), it will be important to continue assessing children and parents' mental health from a long‐term perspective.

In conclusion, the pandemic has created a crisis of unprecedented proportions for stress researchers and the current study is one of the first to assess the mental health changes in family members by using measures prior to the crisis in Canadian parents and children conjointly. Our findings suggest that the first month of the pandemic was a period of heightened stress and anxiety in parents, but not in their children who used more adaptative emotion regulation strategies during the time when they were at home with their parents and away from the personal and social challenges imposed by the school system.

## CONFLICT OF INTEREST

The author declares that there is no conflict of interest that could be perceived as prejudicing the impartiality of the research reported.

## Supporting information

Supplementary Material

## Data Availability

The anonymized data that support the findings of this study, along with scripts, figures and supplemental materials are openly available in Open Science Framework at https://osf.io/4jdvw/.
