# Supplementary material for: Stress, anxiety, emotion regulation and social support in parent‐child dyads prior to and during the onset of the COVID‐19 pandemic
Source: Stress Health. 2022 Aug 5:10.1002/smi.3183. Online ahead of print. doi: 10.1002/smi.3183 (PMC9349815; doi:10.1002/smi.3183)
Supplement: Supplementary file 1 — Supplementary Material [file SMI-9999-0-s001.docx]

**Demographic information – Parents****translated in English from the French version*

**1.** Parental link with the child: **☐**mother **☐**father **☐**other

**2.** How many extracurricular activities (structured activity to which your child is registered that is done outside of school hours, e.g.: chess, soccer, dance, painting, singing, etc.) **does** your child participate in a year?__

**3.** How many hours per week in total? ______________

**4.** What are the activities? Please precise if they are competitive or recreational:

a.______________________________________ **☐**competitive **☐**recreational

b.______________________________________ **☐**competitive **☐**recreational
c.______________________________________ **☐**competitive **☐**recreational

d.______________________________________ **☐**competitive **☐**recreational

e.______________________________________ **☐**competitive **☐**recreational

**5.** What is your ethnicity?

❒Aboriginal❒Arabic ❒Asian ❒African ❒Latin American ❒ Caucasian ❒ Other

**6.** What is your civil status?

❒Single ❒Married ❒Common-law ❒Separated ❒Divorced ❒Widowed

**7.** What is your level of education (last degree obtained)? ______________

**8.** What is your current job? _________________________________________

**9.** If you had to rank your professional status in the society, where would you rank it on a scale of 1 to 10 (1 = bottom of society, 10 = top of society)?_________

**10**. On a scale of 1 to 10, how stressful is your job for you (1=not at all stressful, 10=very stressful)?______

**11.** Your yearly family income is (CAD$):

**☐**0-25 000$

**☐**25 000-50 000$

**☐**50 000-75 000$

**☐**75 000-100 000$

**☐**100 000-125 000$

**☐**125 000-150 000$

**☐**150 000$ and more

**12**. What is the education level of the second parent (last degree obtained)?______________

**13.** What is the second parent's current job? __________________________

**14.** Do you have any of these mental health disorders (for which you have a medical diagnosis)?

a. Depression ☐Yes ☐No

b. Bipolar disorder ☐Yes ☐No

c. Anxiety disorder ☐Yes ☐No

d. Schizophrenia ☐Yes ☐No

e. Drug/alcohol abuse ☐Yes ☐No

f. Dementia ☐Yes ☐No

g. Eating disorder ☐Yes ☐No

h. Other: ____________________________

**15.** Has your child been in daycare? ☐Yes ☐No

**16.** Has your child ever experienced academic failure? ☐Yes ☐No

**17.** How many times a week do you have dinner with all members of the family? _____________

**18.** How many hours of free time per week does your child have? (i.e. time when he/she chooses how to spend his/her time) ________________

|  | Yes | No | Which one(s) ? | Since when ? |
| --- | --- | --- | --- | --- |
| **19.** Does your child have any diagnosed physical health conditions? |  |  |  |  |
| **20.** Does your child have any diagnosed mental health conditions? |  |  |  |  |
| **21.** Does your child have any diagnosed learning conditions? |  |  |  |  |

**22.** Is your child currently receiving treatment or therapy? ☐Yes ☐No

**22a.** If yes, which treatment/therapy?_________________________________

**22b.** If yes, how often? _____________________________

**23.** Does your child take medication? **☐**Yes **☐**No

**23a.** If yes, which one(s)? Indicate the frequency for each: ________________

**24.** There is a lot of talk about performance anxiety experienced by youth, but it also exists in adults. On a scale of 1 to 10 (1 being not at all and 10 being extremely), how much performance anxiety do you think you experience in general?________

**25.** On a scale of 1 to 10 (1 being not at all and 10 being extremely), how much of a perfectionist do you think you are?__________

**26.** On a scale of 1 to 10 (1 being rarely and 10 being always), how often do you receive the social support you need in general?_________

**27.** Do you use social media? **☐** Yes **☐** No

**28.** How many days a week do you use social media?

☐Less than 2 days a week ☐3 to 5 days a week ☐More than 5 days a week

**29.** For how many hours per day?

☐Less than an hour ☐Between 1hr and 2hrs. ☐Between 2hrs and 3hrs ☐More than 3hrs
**30.** What social networks do you use?

| **Social media** | **Number of friends/subscribers** |
| --- | --- |
| 1. Facebook **☐** Yes **☐** No | ☐Less than 50 ☐50 to 100 ☐101 to 200 ☐201 to 300 ☐More than 301 |
| 1. Messenger **☐** Yes **☐** No | ☐Less than 50 ☐50 to 100 ☐101 to 200 ☐201 to 300 ☐More than 301 |
| 1. Instagram **☐** Yes **☐** No | ☐Less than 50 ☐50 to 100 ☐101 to 200 ☐201 to 300 ☐More than 301 |
| 1. Twitter **☐** Yes **☐** No | ☐Less than 50 ☐50 to 100 ☐101 to 200 ☐201 to 300 ☐More than 301 |
| 1. Snapchat **☐** Yes **☐** No | ☐Less than 50 ☐50 to 100 ☐101 to 200 ☐201 to 300 ☐More than 301 |
| 1. YouTube **☐** Yes **☐** No | ☐Less than 50 ☐50 to 100 ☐101 to 200 ☐201 to 300 ☐More than 301 |
| 1. Other **☐** Yes **☐** No | ☐Less than 50 ☐50 to 100 ☐101 to 200 ☐201 to 300 ☐More than 301 |

**31.** On a scale of 1 to 10 (1 being very little and 10 being extremely), how much do you think stress has negative effects...

35a. on performance __________

35b. on physical health ________

35c. on mental health _________

**32.** How anxious do you think your child is generally on a scale of 1 to 10 (1 = very little, 10 = extremely)? _________

**33.** Do you agree to share your child's permanent code with us for testing purposes? ☐ Yes, permanent code_____________________ ☐ No

**34.** Which of the following family structure(s) best describes your child's family structure? (Choose one or more answers as needed):

a. Nuclear family (a couple and their biological child(ren))

b. Only child

c. Divorced parents

d. Separated parents

e. Blended family

f. Shared custody

g. Living with one parent

h. Foster care

i. Adoptive family

j. Two parents of the same sex

k. Other

**Demographic information – Child****translated in English from the French version*

**1.** Girl **☐** Boy **☐** Other **☐
2.** ***To be answered by girls only:** At what age did you first have your period? _____(If you haven't yet, write "NO".)

**3.** Do you use social media  ? **☐** Yes **☐** Non

**4.** How many days a week do you use social media?

☐Less than 2 days a week ☐3 to 5 days a week ☐More than 5 days a week

**5.** For how many hours per day?

☐Less than an hour ☐Between 1hr and 2hrs. ☐Between 2hrs and 3hrs ☐More than 3hrs
**6.** What social media networks do you use? (Check yes to all the ones you use and select the box that describes how many friends/subscribers you have on each of them) :

| **Social media** | **Number of friends/subscribers** |
| --- | --- |
| 1. Facebook **☐** Yes **☐** No | ☐Less than 50 ☐50 to 100 ☐101 to 200 ☐201 to 300 ☐More than 301 |
| 1. Messenger **☐** Yes **☐** No | ☐Less than 50 ☐50 to 100 ☐101 to 200 ☐201 to 300 ☐More than 301 |
| 1. Instagram **☐** Yes **☐** No | ☐Less than 50 ☐50 to 100 ☐101 to 200 ☐201 to 300 ☐More than 301 |
| 1. Twitter **☐** Yes **☐** No | ☐Less than 50 ☐50 to 100 ☐101 to 200 ☐201 to 300 ☐More than 301 |
| 1. Snapchat **☐** Yes **☐** No | ☐Less than 50 ☐50 to 100 ☐101 to 200 ☐201 to 300 ☐More than 301 |
| 1. YouTube **☐** Yes **☐** No | ☐Less than 50 ☐50 to 100 ☐101 to 200 ☐201 to 300 ☐More than 301 |
| 1. Other **☐** Yes **☐** No | ☐Less than 50 ☐50 to 100 ☐101 to 200 ☐201 to 300 ☐More than 301 |

**8.** What do you do when you use social media?

a. Talk with my friends in private ☐ Yes ☐ No

b. Comment on posts that contain a written message (text) ☐ Yes ☐ No

c. Comment on photos ☐ Yes ☐ No

d. Watch videos ☐ Yes ☐ No

e. Identify my friends ☐ Yes ☐ No

f. Post "stories" to show what I am doing ☐ Yes ☐ No

g. Watch what my friends are doing ☐ Yes ☐ No

h. Meet new friends ☐ Yes ☐ No

i. Other ☐ Yes ☐ No

**9.** Approximately how many hours do you sleep per night? ________

**9a.** Do you ever have trouble falling asleep? ☐Never ☐Sometimes ☐Often

**9b.** Do you ever wake up several times a night? ☐Never ☐Sometimes ☐Often

**10.** How many times a week do you do physical activity? __________

**11.** Do you listen to music? _______

**11a.** How many times a week ? ________
**12.** In what context do you listen to music?

a. alone, with headphones on ☐ Yes ☐ No

b. at home, with my parents ☐ Yes ☐ No

c. in the car, with my parents ☐ Yes ☐ No

d. with my friends ☐ Yes ☐ No

**13.** How much do you think your parents can help you manage your stress?

☐Not at all ☐A little ☐A lot

**14.** Do you think your parents' stress sometimes spills over to you? **☐** Yes **☐** No

**15.** In general, how stressed are you on a scale from 1 to 10? (1=Very little, 10=Extremely)_____

**16.** Today, in particular, how stressed do you feel on a scale from 1 to 10 ? (1=Very little, 10=Extremely)_____

**17.** What stresses you out the most : (choose only one answer):

**☐**Exams **☐**Conflicts with your friends **☐**Conflicts with your family

**18.** In general, how anxious are you on a scale from 1 to 10? (1=Very little, 10=Extremely)

**19.** On a scale of 1 to 10, how negative do you think stress is…

**19a.** on performance? (1=Very little, 10=Extremely) _____

**19b.** on physical health? (1=Very little, 10=Extremely)_____

**19c.** on mental health? (1=Very little, 10=Extremely)______

**COVID-19 Questionnaire - Parents**

In recent months, we have been witnessing the evolution of the transmission of the coronavirus COVID-19, originating from the region of Wuhan, in China. Today, as the virus has reached international soil, we would like to know if this situation has worried or still worries you. To that end, please indicate:

**Including you, who is currently living in your household? (If you live alone, please indicate 0 for all choices)**

**Category Number**

Spouse

Parents

Child less than 18 years of age

Child more than 18 years of age

Roommate

Member of extended family

Pet

Others

**Do you belong to one or more of the following groups?**

Older than 70 years old

Health worker

Essential services worker

Working with the public

Traveled outside of the country in 2020

Pre-existing medical condition

+ If you have indicated “yes” to one or more of these categories, please explain the situation briefly

**Amongst those who live with you, how many of them consider themselves to be part of the following groups?**

Older than 70 years old

Health worker

Essential services worker

Working with the public

Traveled outside of the country in 2020

Pre-existing medical condition

+ If you have indicated “yes” to one or more of these categories, please explain the situation briefly

**Are you or have you been in one of the following conditions since the beginning of the crisis?**

| **Condition** | **Choice** |
| --- | --- |
| Quarantine (mandatory isolation, for example: when returning from a trip) | Less than 2 weeks/2 weeks (14 days)/More than 2 weeks |
| Voluntary isolation (you have decided to isolate yourself to avoid transmitting the virus/to be transmitted | Less than 2 weeks/2 weeks (14 days)/More than 2 weeks |

**How has the situation of voluntary isolation or quarantine affected your daily life, both positively and negatively?**

**If you have to work from home, how would you evaluate your ability to perform the tasks that are expected of you in your current environment?**

0 (Definitely not able to meet to expectations)

5 (Meets expectations adequately)

10 (Definitely surpasses expectations)

**At what frequency do you have discussions concerning the COVID-19?**

A few times per week

1x/day

2 to 5 times per day

6 to 9 times per day

10 times and more per day

**Since the arrival of COVID-19 in your city/region, have you experienced any symptoms that may be similar to the symptoms of COVID-19, regardless of the cause of these symptoms?**

0 – No symptoms/10 – Many symptoms

0 – No impact on my functioning/10 – Major impact on my functioning

**Do you voluntarily follow the news related to the COVID-19 pandemic? (Read the newspaper, check the news on your phone, watch the news on TV)?**

Yes/No

**At what frequency do you consult each of the following news sources or means of communication?**

Traditional media (newspaper, radio, television, etc.)

Social networks news feeds (Facebook, Twitter, etc.)

Websites (LaPresse+, Journal de Montreal, etc.)

**How worried or stressed are you right now?**

1 – Not at all….................................................... 1

2…........................................................................ 2

3…........................................................................ 3

4 – Moderately…............................................... 4

5…........................................................................ 5

6…........................................................................ 6

7 – A lot …..................................................... 7

**In your opinion, at what point is this stress attributable to coronavirus?**

1 – Not at all attributable …................................... 1

2…........................................................................ 2

3…........................................................................ 3

4 – Moderately attributable….............................. 4

5…........................................................................ 5

6…........................................................................ 6

7 – Very attributable…..................................... 7

**In the context of this pandemic, how worried are you about:**

|  | **1**  **Not at all worried** | **2** | **3** | **4**  **Moderately worried** | **5** | **6** | **7**  **Excessively worried** |
| --- | --- | --- | --- | --- | --- | --- | --- |
| Your health |  |  |  |  |  |  |  |
| The health of your child(ren) |  |  |  |  |  |  |  |
| The health of one of your loved ones at risk |  |  |  |  |  |  |  |
| The health of one of your loved ones that is not at risk |  |  |  |  |  |  |  |
| Maintaining your employment or your studies (or that of your spouse) or your financial situation? |  |  |  |  |  |  |  |
| Not being able to get the right supplies (e.g. toilet paper, medications, fruits, bread) |  |  |  |  |  |  |  |

Others, specify:

**Normally (before the pandemic), were you generally worried or stressed?**

1 – Not at all….................................................... 1

2…........................................................................ 2

3…........................................................................ 3

4 – Moderately…............................................... 4

5…........................................................................ 5

6…........................................................................ 6

7 – A lot …..................................................... 7

**According to you, stress is generally:**

1 –Very positive....................................................... 1

2........................................................................... 2

3........................................................................... 3

4 – Neither positive nor negative............................4

5........................................................................... 5

6........................................................................... 6

7. – Very negative ................................................7

**COVID-19 Questionnaire – Youth**

In recent months, the coronavirus has been spreading around the world. It is now here in Quebec and we would like to know if this situation has worried you or still worries you.

**Right now, what are your three biggest stressors (things that stress you out)? Place them in order of importance for you (1 being the most stressful)**

**Including you, who currently lives in your home?**

**Category Number**

Mother

Father

Child less than 18 years of age

Child more than 18 years of age

Member of extended family

Pet

Others

**Are you or have you been in one of the following conditions since the beginning of the crisis?**

| **Condition** | **Choice** |
| --- | --- |
| Quarantine (mandatory isolation, for example: when returning from a trip) | Less than 2 weeks/2 weeks (14 days)/More than 2 weeks |
| Voluntary isolation (you have decided to isolate yourself to avoid transmitting the virus/to be transmitted | Less than 2 weeks/2 weeks (14 days)/More than 2 weeks |

**How has the situation of voluntary isolation or quarantine affected your daily life, both positively and negatively?**

**At what frequency do you have discussions about the coronavirus?**

A few times per week, 1x/day, 2 to 5x per day, 6 to 9xper day, 10x and more per day

**Since the arrival of the coronavirus in Quebec (February 28^th^, 2020), have you had symptoms (manifestations) that resemble those of the coronavirus (cough, fever, muscular pains, sore throat), even if they were not caused by the coronavirus?**

0 – No symptoms/10 – Many symptoms

0- It hasn’t affected me at all /10- It has greatly affected me

**Do you voluntarily follow the news related to the coronavirus? (Read the newspaper, check the news on your phone, watch the news on TV)?**

Yes/No

**At what frequency do you consult each of the following news sources or means of communication? (How many hours per day)**

Traditional media (newspaper, radio, television, etc.)

Social networking news feeds and websites (Facebook, Twitter, LaPresse+, etc.)

**How worried or stressed are you right now?**

1 – Not at all….................................................... 1

2…........................................................................ 2

3…........................................................................ 3

4 – Moderately…............................................... 4

5…........................................................................ 5

6…........................................................................ 6

7 – A lot …..................................................... 7

**In your opinion, how much of this stress is caused by the coronavirus?**

1 – Not at all attributable …................................... 1

2…........................................................................ 2

3…........................................................................ 3

4 – Moderately attributable….............................. 4

5…........................................................................ 5

6…........................................................................ 6

7 – Much attributable…..................................... 7

**In the context of this pandemic (the coronavirus that is spreading throughout the world), how worried are you about:**

|  | **1**  **Not at all worried** | **2** | **3** | **4**  **Moderately worried** | **5** | **6** | **7**  **Excessively worried** |
| --- | --- | --- | --- | --- | --- | --- | --- |
| Your health? |  |  |  |  |  |  |  |
| The health of your parents? |  |  |  |  |  |  |  |
| The health of a person that matters to you? |  |  |  |  |  |  |  |
| The rest of your school year? |  |  |  |  |  |  |  |
| Your parent's jobs or your job? |  |  |  |  |  |  |  |
| To be missing something (e.g. toilet paper, medications, fruits, bread)? |  |  |  |  |  |  |  |

Others, specify:

**Normally (before the virus arrived in Quebec), are you generally worried or stressed?**

1 – Not at all….................................................... 1

2…........................................................................ 2

3…........................................................................ 3

4 – Moderately…............................................... 4

5…........................................................................ 5

6…........................................................................ 6

7 – A lot …..................................................... 7

**According to you, stress is generally:**

1 –Very positive....................................................... 1

2........................................................................... 2

3........................................................................... 3

4 – Neither positive nor negative......................... 4

5........................................................................... 5

6........................................................................... 6

7 – Very negative ...................................................... 7

**Table 1. Estimated marginal means of mental health, emotion regulation strategies and social support measures for parents and children across time and by sex**

|  | **Pre-pandemic measures**  **(2019)** | | | | | | | | |  | **Onset of the pandemic measures (2020)** | | | | | | | | |  |  |
| --- | --- | --- | --- | --- | --- | --- | --- | --- | --- | --- | --- | --- | --- | --- | --- | --- | --- | --- | --- | --- | --- |
|  | **T1** | | | |  | **T2** | | | |  | **T3** | | | | | | | | |  |  |
|  | **Female** | | **Male** | |  | **Female** | | **Male** | |  | | | **Female** | | | | **Male** | |  |  |  |
| **Measure** | ***M*** | ***SD*** | ***M*** | ***SD*** |  | ***M*** | ***SD*** | ***M*** | ***SD*** |  | | | ***M*** | | ***SD*** | | ***M*** | | ***SD*** | | |
| Parents |  |  |  |  |  |  |  |  |  |  | |  | |  | |  | |  | | |  |
| Perceived Stress (PSS) | 19.38 | .83 | 13.53 | 1.53 |  |  |  |  |  |  | | 23.57 | | .78 | | 18.76 | | 1.44 | | |  |
| State Anxiety (STAI-Y1) | 30.19 | .86 | 26.61 | 1.60 |  |  |  |  |  |  | | 37.12 | | 1.18 | | 31.89 | | 2.20 | | |  |
| Anxiety Sensitivity (ASI) | 11.58 | .69 | 9.46 | 1.30 |  |  |  |  |  |  | | 13.93 | | .90 | | 9.54 | | 1.68 | | |  |
| Cognitive Reappraisal (ERQ) | 30.49 | .79 | 25.93 | 1.48 |  |  |  |  |  |  | | 31.15 | | .74 | | 27.21 | | 1.38 | | |  |
| Emotional Suppression (ERQ) | 11.77 | .54 | 13.46 | 1.01 |  |  |  |  |  |  | | 12.21 | | .50 | | 12.57 | | .94 | | |  |
| Children |  |  |  |  |  |  |  |  |  |  | |  | |  | |  | |  | | |  |
| Perceived Stress (PSS-C) ^†^ | 14.51 | .66 | 11.63 | .83 |  | 13.31 | .64 | 10.53 | .81 |  | | 14.73 | | .58 | | 12.49 | | .74 | | |  |
| State Anxiety (STAI-C) ^†^ | 33.21 | .78 | 28.81 | .98 |  | 32.82 | .72 | 28.99 | .90 |  | | 33.88 | | .75 | | 29.48 | | .95 | | |  |
| Anxiety Sensitivity (CASI) ^†^ | 31.52 | .95 | 28.66 | 1.11 |  | 31.56 | .92 | 27.18 | 1.08 |  | | 31.33 | | .94 | | 26.67 | | 1.09 | | |  |
| Cognitive Reappraisal (ERQ-CA) ^†^ | 19.83 | .52 | 20.82 | .65 |  | 21.11 | .51 | 19.94 | .63 |  | | 20.11 | | .53 | | 19.38 | | .66 | | |  |
| Emotional Suppression (ERQ-CA) ^†^ | 12.15 | .36 | 11.94 | .45 |  | 11.70 | .42 | 11.43 | .52 |  | | 11.28 | | .41 | | 10.65 | | .52 | | |  |
| Co-rumination (CRQ) ^†^ |  |  |  |  |  | 29.48 | .84 | 28.70 | 1.06 |  | | 27.21 | | .91 | | 24.62 | | 1.16 | | |  |
| Social support – friends(CASSS-fr) ^†^ | 58.73 | 1.30 | 54.99 | 1.68 |  | 59.66 | 1.28 | 58.23 | 1.64 |  | | 59.87 | | 1.21 | | 54.01 | | 1.56 | | |  |
| Social support – parents (CASSS-pr) ^†^ | 58.09 | 1.08 | 58.41 | 1.37 |  | 58.75 | 1.06 | 59.30 | 1.33 |  | | 58.23 | | 1.31 | | 58.28 | | 1.65 | | |  |
| Social support – teachers (CASSS-te) ^†^ | 57.92 | 1.24 | 58.54 | 1.46 |  | 59.38 | 1.16 | 59.30 | 1.36 |  | | 59.39 | | 1.41 | | 58.08 | | 1.66 | | |  |

*Notes:* p<.01

**Table 2. Significant at 0.01 level Pearson correlations between children’s and parents’ outcomes at pre-pandemic measures and during the onset of the pandemic**

|  | **Pre-pandemic measures (2019)** | | | | | | | | | |  | |  | **Onset of the pandemic measures (2020)** | | | | | | | | |  |  |
| --- | --- | --- | --- | --- | --- | --- | --- | --- | --- | --- | --- | --- | --- | --- | --- | --- | --- | --- | --- | --- | --- | --- | --- | --- |
| **Measure** | ***1*** | ***2*** | ***3*** | ***4*** | ***5*** | ***6*** | ***7*** | ***8*** | ***9*** | ***10*** | |  | | ***1*** | ***2*** | ***3*** | ***4*** | ***5*** | ***6*** | ***7*** | ***8*** | ***9*** | | ***10*** |
| Parents |  |  |  |  |  |  |  |  |  |  | |  | |  |  |  |  |  |  |  |  |  | |  |
| 1. Perceived Stress (PSS) |  | .64 | .27 |  |  |  |  |  |  |  | |  | |  | .59 | .45 |  |  |  |  |  |  | |  |
| 2. State Anxiety (STAI-Y1) |  |  |  |  |  |  |  |  |  |  | |  | |  |  | .45 | -.29 |  |  |  |  |  | |  |
| 3. Anxiety Sensitivity (ASI) |  |  |  |  | .22 |  |  |  |  |  | |  | |  |  |  |  | .29 |  |  |  |  | |  |
| 4. Cognitive Reappraisal (ERQ) |  |  |  |  |  |  |  |  |  |  | |  | |  |  |  |  |  |  |  |  |  | |  |
| 5. Emotional Suppression (ERQ) |  |  |  |  |  |  |  |  |  |  | |  | |  |  |  |  |  |  |  |  |  | |  |
| Children |  |  |  |  |  |  |  |  |  |  | |  | |  |  |  |  |  |  |  |  |  | |  |
| 6. Perceived Stress (PSS-C) |  |  |  |  |  |  | .64 | .57 | -.23 | .34 | |  | |  |  |  |  |  |  | .59 | .31 |  | | .33 |
| 7. State Anxiety (STAI-C) |  |  |  |  |  |  |  | .55 |  | .24 | |  | |  |  |  |  |  |  |  | .58 |  | | .47 |
| 8. Anxiety Sensitivity (CASI) |  |  |  |  |  |  |  |  |  |  | |  | |  |  |  |  |  |  |  |  |  | | .27 |
| 9. Cognitive Reappraisal (ERQ-CA) |  |  |  |  |  |  |  |  |  |  | |  | |  |  |  |  |  |  |  |  |  | |  |
| 10. Emotional Suppression (ERQ-CA) |  |  |  |  |  |  |  |  |  |  | |  | |  |  |  |  |  |  |  |  |  | |  |
